# Proteomic discovery and verification of serum amyloid A as a predictor marker of patients at risk of post-stroke infection: a pilot study

**DOI:** 10.1186/s12014-017-9162-0

**Published:** 2017-07-12

**Authors:** L. Azurmendi, V. Lapierre-Fetaud, J. Schneider, J. Montaner, M. Katan, Jean-Charles Sanchez

**Affiliations:** 1 Translational Biomarker Group, Department of Human Protein Sciences, University of Geneva, Rue Michel Servet 1, 1211 Geneve 4, Switzerland; 2 Department of Neurology, University Hospital of Zurich, Zurich, Switzerland; 3 Department of Medicine, Universitat Autònoma de Barcelona, Barcelona, Spain

## Abstract

**Background:**

Post-stroke infections occur in 20–36% of stroke patients and are associated with high morbidity and mortality rates. Early identification of patients at risk of developing an infection could improve care via an earlier treatment leading to a better outcome. We used proteomic tools in order to discover biomarkers able to stratify patients at risk of post-stroke infection.

**Methods:**

The post hoc analysis of a prospective cohort study including 40 ischemic stroke patients included 21 infected and 19 non-infected participants. A quantitative, isobaric labeling, proteomic strategy was applied to the plasma samples of 5 infected and 5 non-infected patients in order to highlight any significantly modulated proteins. A parallel reaction monitoring (PRM) assay was applied to 20 additional patients (10 infected and 10 non-infected) to verify discovery results. The most promising protein was pre-validated using an ELISA immunoassay on 40 patients and at different time points after stroke onset.

**Results:**

Tandem mass analysis identified 266 proteins, of which only serum amyloid A (SAA1/2) was significantly (*p* = 0.007) regulated between the two groups of patients. This acute-phase protein appeared to be 2.2 times more abundant in infected patients than in non-infected ones. These results were verified and validated using PRM and ELISA immunoassays, which showed that infected patients had significantly higher concentrations of SAA1/2 than non-infected patients at hospital admission, but also at 1, 3, and 5 days after admission.

**Conclusions:**

The present study demonstrated that SAA1/2 is a promising predictor, at hospital admission, of stroke patients at risk of developing an infection. Further large, multicenter validation studies are needed to confirm these results. If confirmed, SAA1/2 concentrations could be used to identify the patients most at risk of post-stroke infections and therefore implement treatments more rapidly, thus reducing mortality.

**Electronic supplementary material:**

The online version of this article (doi:10.1186/s12014-017-9162-0) contains supplementary material, which is available to authorized users.

## Background

Stroke is a serious medical condition produced by brain cell death. It occurs when there is a lack of blood flow to the brain (~80% of cases) or a hemorrhage affecting the brain or its surroundings (20%). Every year, around 15 million people will suffer a stroke, leading to 6 million deaths and 5 million disabled patients [1–3]. Around 40% of patients die within the first weeks following the stroke [4, 5]. Non-modifiable factors, such as the severity of the stroke or the age of the patient, are highly correlated with mortality [6, 7]. However, one-third of deaths result from potentially preventable stroke-associated complications. Nosocomial infection, particularly bacterial pneumonia, is the most common complication after stroke, with an incidence of 5–22% [8–10]. Despite the intensive care given to these patients, infection rates remain elevated and are associated with bad functional outcome and mortality [11]. The high incidence of infection is likely to be a result of an impaired immune function. The patient’s reduced ability combat bacteria is a consequence of the initial brain damage [12, 13]. Therefore, the early identification of patients who might be prone to developing an infection after stroke is a necessary step towards better hospital management, more rapid implementation of treatments, and improved long-term patient outcomes [13, 14].

In clinical practice, the diagnosis of post-stroke infection is a challenging one as there has been no satisfactory concordance between different studies. The most widely studied markers of post-stroke infection—procalcitonin (PCT), C-reactive protein (CRP), and white blood cells (WBC)—have only shown moderate predictive value, and their levels do not increase early enough to be of help before the infection is clinically apparent [15, 16]. Clinical signs such as older age, fever, severe stroke, or dysphagia, among others, have been linked to post-stroke associated pneumonia [17]. Nevertheless, they are not specific enough to act as individual markers. Using a combination of these markers with clinical scales such as the A2DS2, AIS-APS, and ISAN [17–19] have not been applied routinely in clinical practice. The gold standard for diagnosing an infection is the result from a bacterial culture, yet this may take 2 days. All of these reasons can lead to antibiotic treatment being started too late, with the unfortunate associated consequences. There is thus evidence of a need for a reliable early biomarker [20].

The present study aimed to use proteomic approaches to find a biomarker that could be tested for at hospital admission in order to identify patients at risk of developing a post-stroke infection. To do this, we investigated the plasma proteomes of infected and non-infected patients, using isobaric labeling methods. After selecting SAA1/2 as the most promising protein, parallel reaction monitoring (PRM) and the enzyme-linked immunosorbent assays (ELISA) confirmed its ability to predict which patients were at risk of infection after a stroke.

## Methods

### Study design and setting

We performed a post hoc analysis of a prospective cohort study which included 40 ischemic stroke patients (ClinicalTrials.gov.NCT00390962) who had been hospitalized consecutively at the University Hospital of Basel (Switzerland) between November 2006 and November 2007. The study protocol was conducted according to the principles expressed in the Declaration of Helsinki and with the approval of the local ethics committee. Before enrolment, informed consent was obtained from patients, their relatives, or their legal guardians.

### Clinical protocol

Comprehensive information on the assessment of the study participants’ demographic and vascular risk factors has been published previously [15]. Briefly, ischemic stroke was defined according to the World Health Organization criteria [21]. A detailed history was obtained for vascular risk factors, vital signs, and relevant comorbidities as assessed using the Charlson Comorbidity Index (CCI) medication taken prior to the stroke. Neurological deficits were estimated using the National Institutes of Health Stroke Scale (NIHSS). Patients underwent the following standardized diagnostic workup: brain computer tomography (CT) and/or magnetic resonance imaging, long-term electrocardiography, echocardiography, and neurosonographic imaging of the extracranial and intracranial arteries. Stroke etiology was determined according to the TOAST (Trial of Org 10172 in Acute Stroke Treatment) classification criteria, which distinguish large-artery arteriosclerosis, cardio embolism, small-artery occlusion, other etiologies, and undetermined etiologies [22].

### Definition of stroke-associated Infections

Stroke-associated infection (SAI) was defined as any infection occurring within the first 5 days after hospital admission [13]. Infections were diagnosed according to the U.S. Centers for Disease Control and Prevention (CDC) criteria [15]. We distinguished between pneumonia, urinary tract infection (UTI), and other infections (OI). Pneumonia was diagnosed when at least one symptom from each of the two following symptom groups was present: (1) abnormal respiratory examination, pulmonary infiltrates in chest X-rays; (2) productive cough with purulent sputum, positive microbiological cultures from the lower respiratory tract or blood cultures.

Diagnosis of a UTI required two of the following criteria to be met: fever (≥38.0 °C), urine sample positive for nitrite, leukocyturia (≥40/µL), or significant bacteriuria (≥10^4^/mL of an uropathogen). OI were diagnosed if white blood cell count was ≥11,000/mL and CRP was ≥10 mg/L or temperature was ≥38.0 °C and an infectious manifestation was present. The treating physician made the diagnosis of pneumonia during hospitalization. This was then validated post hoc using charts.

The time point of diagnosis was taken to be the beginning of clinical symptoms which led to the diagnostic workup and resulted in the diagnosis of infection. In order to exclude any acute infections that had preceded the stroke, patients with an admission temperature >38 °C, reporting an infection lasting up to 3 days before the onset of stroke, or who required mechanical intubation were not included in the study.

### Blood sample collection

Blood samples were collected from venous blood puncture within the first 72 h following symptom onset and then 1, 3, and 5 days after admission. Blood was centrifuged for 30 min at 3000×*g*, collected in EDTA, tubes and stored at −80 °C.

### Proteomic study

#### Quantitative proteomic analysis: TMT

Quantitative proteomic analyses were performed on five infected and five non-infected patients at hospital admission. The aim was to identify significantly regulated proteins between the two groups in order to find a promising infection marker.

##### Reduction, alkylation, digestion, and TMT labeling

The quantitative proteomic experiment used 1 µL of each plasma sample. These amounts were dried and reconstituted in 16.6 µL of 6 M urea in tetraethylammonium bromide (TEAB) 0.1 M. The proteins were reduced by adding 1 µL of 50 mM tris-(2-carboxyethyl) phosphine hydrochloride (TCEP) to each sample, and they then reacted for 1 h at 37 °C. After the sample had cooled to room temperature, the alkylation step required mixing the solution with 1 µL of iodoacetamide 400 mM and storage at room temperature for 30 min. Sixty seven µL of TEAB 0.1 M were added to reduce the concentration of urea to <2 M. The digestion was carried out overnight at 37 °C using 1 µg of trypsin for each 20 µg of protein. The protocol is detailed by Dayon et al. [23, 24].

Subsequently, each digested plasma sample was labeled with one of the 10 TMT reagents (Thermo Fisher Scientific, Waltham, USA). Infected patients’ samples were labeled with TMTs 127n, 128n, 129n, 130n, and 131n. Non-infected patients’ samples were labeled with TMTs 126, 127c, 128c, 129c, and 130c. To calculate experimental error, 1 µg of β-lactoglobulin was spiked in each sample. All the samples were pooled, desalted using a C18 Macro SpinColumn, and dried in a speed-vacuum.

##### Off-gel electrophoresis (OGE)

OGE separation was carried out using an Agilent 3100 Off-Gel fractionator, as per the manufacturer’s instructions. Previously dried samples were reconstituted using the OGE solution and then focused using an immobilized pH gradient (IPG) dry strip (13 cm, pH 3–10) [23]. After OGE, samples were desalted using a C18 Micro SpinColumn, dried in the speed-vacuum, and stored at −20 °C until analysis.

##### LC–MS/MS

A Q-Exactive Plus mass spectrometer (ThermoFisher, San Jose, CA), coupled with nanoflow high-pressure liquid chromatography (HPLC), was used to analyze the OGE fractions, as previously described [25]. Briefly, peptides reconstituted using 5% CAN, 0.1% FA, were trapped in a 5 µm 200 Å Magic C18 AQ (Michrom) 0.1 × 20 mm pre-column and separated in a 5 µm 100 Å Magic C18 AQ (Michrom) 0.75 × 150 mm column with a gravity-pulled emitter. Both columns were made in-house. The analytical separation ran for 65 min using a gradient of H_2_O/FA 99.9/0.1% (solvent A) and CH_3_CN/FA 99.9/0.1% (solvent B). The gradient ran at a flow rate of 220 nL/min as follows: 0–1 min 95% A and 5% B, then to 65% A and 35% B at 55 min, and 20% A and 80% B at 65 min. For the MS survey scans, OT resolution was set to 60,000 and the ion population was set to 5 × 105 with an m/z window from 400 to 2000. A maximum of three precursors were selected for both collision-induced dissociation (CID) in the LTQ and higher-energy collisional dissociation (HCD) with analysis in the OT. For MS/MS in the LTQ, the ion population was set to 7 × 10^3^ (isolation width of 2 m/z), whereas for MS/MS detection in the OT, it was set to 2 × 10^5^ (isolation width of 2.5 m/z), with a resolution of 7500, a first mass at m/z = 100, and a maximum injection time of 750 ms. The normalized collisional energies were set to 35% for CID and 60% for HCD.

Protein identification MS data were processed using EasyProtConv. Peak lists were obtained using the 12 OGE fractions and the combination of HCD-CID raw data peak lists were generated. Afterwards, these data were submitted to an EasyProt software platform (version 2.3, build 718) that uses Phenyx software (GeneBio, Geneva, Switzerland) for protein identification. The protein search was made using the Uniprot/Swiss-Prot database (2014–10, 669903) [26], applying the following search criteria: *Homo* *sapiens* taxonomy, oxidized methionine (as the variable modification), and cysteine carbamethylation, TMT^10^ lysine, and TMT^10^ amino-terminus (as the fixed modifications). Trypsin was selected as the proteolytic enzyme, allowing one missed cleavage. Parent-ion tolerance was set to 10 ppm and the accuracy of fragment ions to 0.6 Da. Only proteins with a less than 1% false discovery rate (FDR) and at least two different unique peptides were selected for further analysis [27]. A minimum peptide length of 6 amino acids was used.

Protein quantification used the Isobar R package [28]. The manufacturer’s isotopic distribution data was used to correct the isotopic impurities of TMT^10^ reporter-ion intensities. The equal median intensity method was used to normalize the reporter intensities. Peptides which did not present reporter intensities were not quantified. The infection/no infection ratio was calculated for each peptide, combining the reporter-ion intensities between infected patient channels (127n, 128n, 129n, 130n, and 131n) and non-infected patient channels (126, 127c, 128c, 129c, and 130c). To test the ratio’s accuracy and biological significance, technical and biological variability were calculated for each protein ratio. A ratio *p* value and sample *p* value were calculated for each variable. Furthermore, only proteins with a cut-off threshold value higher than 1.5 or lower than 0.67 were considered [29–31].

#### SAA1/2 PRM analysis

Parallel reaction monitoring (PRM) analysis was performed on ten infected and ten non-infected plasma samples using a Q-Exactive Plus mass spectrometer (ThermoFisher), as previously described [32]. The aim was to verify the discovery results.

Each sample was loaded into a PepMap precolumn (2 cm × 75 µm i.d., C18, 3 µm, and 100 Å pore size). Subsequent separation was performed in a PepMap column (50 cm × 75 µm i.d., C18, 2 µm, 100 Å pore size). A mixture of mobile A and B phases was used for peptide elution. The phase A solvent was composed of 0.1% (v/v) formic acid (Biosolve) and HPLC-grade water (Romil); the phase B solvent was composed of 0.1% (v/v) formic acid in HPLC-grade acetonitrile (Romil). To perform the separation, a linear gradient of 5–35% solvent B at 250 nL/min for 60 min was set and it was followed by a washing step (35–90% of solvent B for 10 min).

Three masses were targeted (doubly and triply charged ions), corresponding to total SAA, but also specifically to SAA1 and SAA2. The selection of the different peptides was performed considering two different criteria: a previous SAA PRM study and the results of our quantitative proteomic analysis [32]. The three peptides selected in this way were tryptic peptides associated to each isoform.

This inclusion list triggered targeted scans at a resolving power of 70,000, with an isolation width of 1 Th around the m/z of interest, an AGC target of 1 × 10^6^, a maximum injection time of 100 ms, and a normalized collision energy of 27% in a higher-energy c-trap dissociation (HCD) cell.

##### Data analysis

Data were analyzed using the targeted MS/MS feature available in Skyline v3.5 software [33]. In order to confirm the identity of the peptides, a data dependent acquisition spectral library of annotated reference MS/MS spectra was created from the two pools of plasma samples composed of infected and non-infected patients. Peptides were quantified by extracting the peak areas of accurate fragment ions (<6 ppm), and they were then integrated across the peptides’ elution profiles. For each peptide, transition peak areas were normalized by the average of the sum of the transition peak areas for all the peptides across the runs.

#### SAA1/2 ELISA measurement

The Vascular Injury Panel-I electrochemiluminescence (ECL) assay was used to determine the levels of SAA1/2 in 40 stroke patients, as per the manufacturer’s instructions (Meso Scale Discovery, Gaithersburg, MD). Each plasma sample was diluted 1:1000 with using sample diluent provided by the kit. An ECL detection system using multi-array technology (SECTOR Imager 2400, Meso Scale Discovery) was used to determine analyte concentrations. Samples were measured in a single detection.

#### Statistical analyses

Statistical analyses were carried out using SPSS software (v21, SPSS Inc., Chicago, IL). Analytes were not normally distributed, so the Mann–Whitney U-test was used to compare the two unpaired groups. Fisher’s exact test and the Chi squared test were used to assess whether patients with and without infection were significantly different according to their gender, medical history, clinical data, laboratory values, lesion size, or TOAST. All statistical tests were two-tailed, and a p value <0.05 was considered statistically significant.

Multivariate analyses were performed to assess the associations between variables. The presence/absence of infection was set as the dependent variable, and SAA, CRP, WBC, and NIHSS were set as confounders. The model was validated using the bootstrap method. Categorical data were dichotomized according to the criteria in the table of demographic characteristics. Longitudinal data were also dichotomized according to the best cut-off obtained from area under the receiver operating characteristic (ROC) curve (AUC) analysis.

## Results

### Baseline population characteristics

Of 40 consecutively enrolled ischemic stroke patients, 21 developed an infection within 5 days of stroke onset (day 4 was the median day of infection development after the cerebrovascular event). Mean patient age was 79 years old (IQR: 70–82 years) and 55% of patients were men. Patients with severe strokes, resulting in higher NIHSS values at hospital admission, were more prone to developing an infection than patients with minor strokes. Other factors, such as hypertension, diabetes mellitus, or smoking, did not significantly affect the development of an infection. Nevertheless, according to the modified Rankin Scale, patient outcome appeared to be significantly affected by the development of an infection, as most of the patients with a poor outcome had developed an infection during their hospital stay.

At hospital admission, levels of WBC and CRP were within the normal range in both groups, with no significant differences found between infected and non-infected patients. Patients’ demographic characteristics are summarized in Table 1.

**Table 1:** Baseline data

	All patients	No infection	Infection	*p* value
	(*n* = 40)	(*n* = 19)	(*n* = 21)	
Demographic data				
Age, median (IQR)	79.2 (70.4–82)	78.3 (74–80.5)	80.4 (69.5–83)	0.78
Female sex, *n* (%)	18 (45)	9 (47.4)	9 (42.9)	1
Medical history, *n* (%)				
Hypertension	31 (77.5)	12 (63.2)	19 (90.5)	0.06
Atrial fibrillation	9 (22.5)	3 (15.8)	6 (28.6)	0.17
Current smoking	11 (27.5)	5 (26.3)	6 (28.6)	0.64
Diabetes mellitus	7 (17.5)	4 (21.1)	3 (14.3)	0.69
Coronary heart disease	10 (25)	4 (21.1)	6 (28.6)	0.72
Previous stroke	11 (27.5)	5 (26.3)	6 (28.6)	1
Clinical data, median (IQR)				
NIHSS at admission	5.5 (2–12)	3 (2–7)	12 (4–14)	0.01
Laboratory values, median (IQR)				
WBC (g/l)	8.6 (6.8–10.1)	7.7 (6.2–9.3)	9.3 (7.4–11.2)	0.14
CRP (mg/l)	3.6 (3–9.1)	3.6 (3–6.8)	4.8 (3–17.4)	0.22
Lesion size on MR, DWI^b^				
Small (1–10 mm^3^)	23 (27.5)	13 (68.4)	10 (47.6)	0.47
Medium (10–100 mm^3^)	8 (20)	3 (15.8)	5 (23.8)	0.75
Large (>100 mm^3^)	1 (2.5)	0 (0)	1 (4.8)	0.69
TOAST				
Large vessel stroke	8 (20)	3 (15.8)		0.69
Cardioembolic stroke	8 (20)	4 (21.1)		1
Microangipathic stroke	14 (35)	5 (26.3)		0.33
Other	0 (0)	0 (0)		
Unknown	10 (25)	7 (36.8)		0.15

### Proteomic results

In order to find a biomarker able to distinguish, at hospital admission, which patients will and will not develop a post-stroke infection, the proteomes of five infected and five non-infected stroke patients were compared using quantitative proteomic analysis. Applying the criteria of a maximum of 1% FDR and at least two unique peptides, 266 proteins were quantified (Additional file 1: Table 1). Of all the proteins, serum amyloid A1 appeared to be the only significantly (*p* = 0.007) regulated protein between the two groups of patients, with a ratio of 2.2 after Bonferroni correction.

**Fig. 1:**
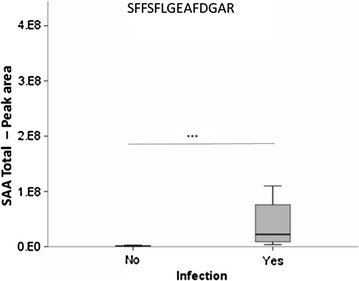
Ratio of SFFSFLGEAFDGAR peptide abundance between patients who went on to develop an infection and those who did not. The SFFSFLGEAFDGAR peptide is common to all the different isoforms of SAA

To verify the results obtained by the TMT^10^ plex during the discovery phase, a further PRM analysis was performed on a new batch of patients. Consequently, we targeted three transitions of the tryptic SFFSFLGEAFDGAR peptide in 10 infected and 10 non-infected patients. This peptide is common to all the different isoforms of acute-phase SAA. By measuring its concentration, therefore, we were sure to measure the total amount SAA present in blood and not only that of one of the different described isoforms. As shown in Fig. 1, the concentration of SFFSFLGEAFDGAR was significantly higher (p < 0.001) in infected patients than in non-infected ones, confirming that there was a clear over-production of SAA in patients who went on to develop an infection.

### Different SAA isoforms for infection development

Further PRM analyses were performed on the same 20 patients in order to evaluate whether either of the acute phase isoforms (SAA1 and SAA2) had a more significant effect on infection and inflammatory processes. The high sequence-similarity between the SAA1 and SAA2 isoforms prevented an evaluation of their effects using classic ELISAs. The present study measured three transitions in the FFGHGAEDSLADQAANEWGR peptide (unique to SAA1) and GPGGAWAAEVISNAR peptide (unique to SAA2) across 10 infected and 10 non-infected patients. As Additional file 2: Fig. 1 shows, both peptides were significantly (p < 0.001) more abundant in infected patients than in non-infected ones.

### Kinetics of serum amyloid A1/2

**Fig. 2:**
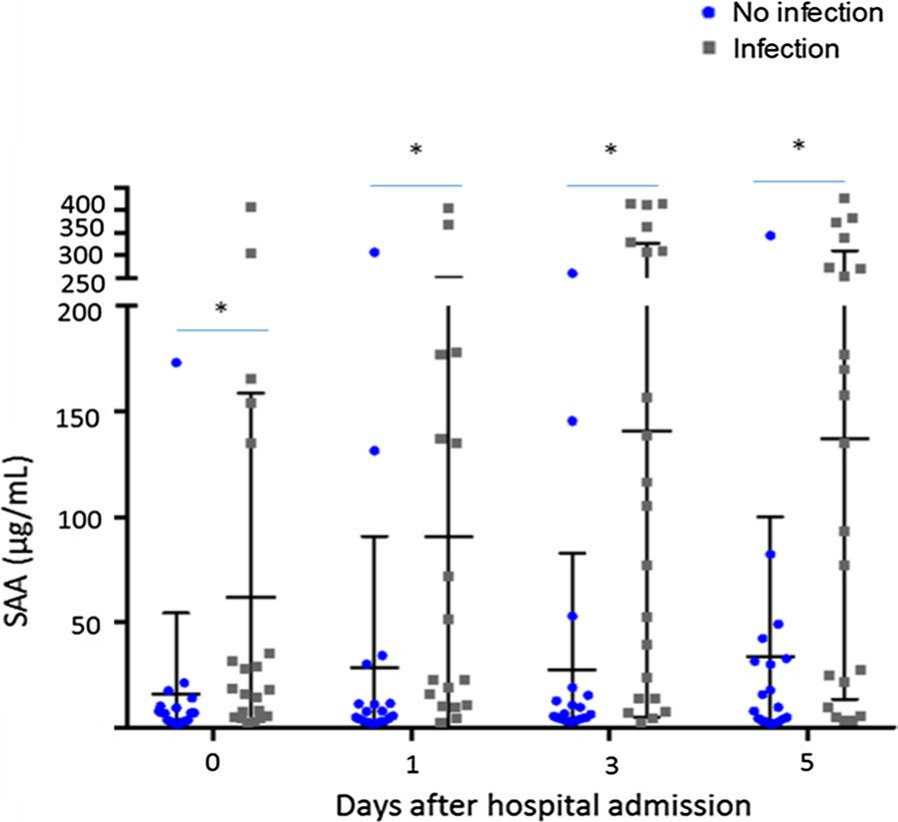
Kinetics of SAA concentrations at hospital admission, 1, 3, and 5 days after stroke onset. Text under Fig. 2 in PDF: SAA concentrations are shown by *grey square* for infected patients and by *blue square* for non-infected patients. Comparisons between the two groups were made using the Mann–Whitney *U* test. *Asterisk* significance level reported after the Bonferroni correction (p < 0.05)

Serum amyloid A1/2 plasma concentrations were subsequently measured in a new group of 21 infected and 19 non-infected patients in order to validate the previous proteomic results. Concentrations of this acute-phase reactant molecule were measured at hospital admission and at 1, 3, and 5 days after hospitalization, using an SAA1/2 ELISA assay. Initially, analyses were performed separately in those patients used for the discovery step and in those used for the verification/validation step. As Additional file 3: Fig. 2 shows, in both cases, SAA concentrations were significantly higher in patients who went on to develop a post-stroke infection than in those who did not. Subsequently, analyses were performed again when all the patients were evaluated together. As Fig. 2 shows, peptide concentrations were again significantly higher in infected patients than in non-infected patients, at all time points, particularly at 3 days (*p* = 0.01) and 5 days (*p* = 0.01) after stroke onset.

SAA measurements were evaluated to distinguish between the two groups of patients at D0, D1, D3, and D5. As Table 2 shows, the accuracy of SAA measurements in distinguishing which patients went on to develop an infection and which did not reached values of 73.2% (cut-off: 14.2 µg/mL) and 77.1% (cut-off: 8.8 µg/mL) at hospital admission and 1 day after, respectively. Three days after hospitalization, the AUC of SAA was slightly better, reaching a value of 80.7% (cut-off: 21.4 µg/mL), and 5 days after hospitalization, the AUC was 76.7% (cut-off: 87.7 µg/mL).

**Table 2:** Capacity of plasma concentrations of SAA to distinguish between patients who went on to develop an infection and those who did not

Day	Number of patients		Mean SAA concentration (µg/mL) ± SD			ROC curve			
	No infection	Infection	No infection	Infection	p value	AUC (95% CI)	Cut-off	SP% (95% CI)	SE% (95% CI)
						pAUC (95% CI)		SP 90–100% (95% CI)	
0	19	21	16 ± 38.4	61.8 ± 96.7	0.01	73.2 (55.9–87)	14.2	84.2 (68.4–100)	61.9 (38.1–81)
						2.53 (0–6.7)	24.7	94.7 (84.2–100)	42.9 (23.8–62.02)
1	19	17	28.2 ± 62.6	57.7 ± 92.9	0.005	77.1 (60.1–92)	8.8	63.2 (42.1–84.2)	88.2 (70.6–100)
						2.3 (0.6–6.5)	133.4	94.7 (84.2–100)	35.3 (11.8–58.8)
3	19	21	27.6 ± 55.2	140.9 ± 136	0.001	80.7 (66.2–93.2)	21.4	84.2 (68.4–100)	71.4 (52.4–90.5)
						3.6 (1.7–7.6)	233.9	100 (100–100)	33.3 (14.3–52.4)
5	19	21	33.6 ± 66.4	137 ± 123.5	0.003	76.7 (61.2–89.7)	87.8	94.7 (84.2–100)	57.1 (38.1–76.2)
						3.5 (0.5–7.6)	87.8	94.7 (84.2–100)	57.1 (38.1–76.2)

**Fig. 3:**
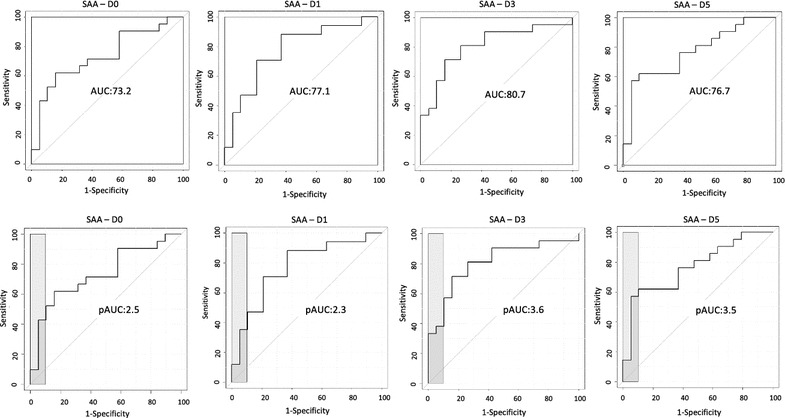
ROC curves for SAA representing the ability to differentiate between patients who went on to develop an infection and those who did not, on the day of hospital admission (D0) and 1 day (D1), 3 days (D3), and 5 days after (D5) stroke onset

To evaluate the capacity of SAA1/2 measurement to rule-in patients at risk of infection, we set specificity (SP) at between 90 and 100%. At hospital admission, with a 94.7% SP, SAA measurement reached 42.9% sensitivity (SE) and a partial AUC of 2.5% (Table 2). Three days after hospitalization, SP reached 100%, SE was 33.3%, and the partial AUC was 3.6% (Table 2). All the AUC and pAUC curves obtained at the different time points are represented in Fig. 3. These AUC and pAUC values were obtained using different cut-off concentrations corresponding to the best combination of SP and SE.

However, due to the high variability of the SAA concentrations obtained, we decided to evaluate the possibility of using a ratio based on those concentrations to predict the development of an infection. As Table 2 shows, patients who went on to develop an infection during their hospital stay, presented with an average 2.4 times greater concentration of SAA on D3 than on D1. For patients who did not become infected, average SAA concentrations remained very similar (ratio of 0.97), with no significant increase, thus suggesting that this ratio could be used as an indicator of patients at risk.

### Multivariate analyses

Finally, we performed multivariate analyses in order to confirm that SAA was a promising biomarker of post-stroke infection and to assess whether it was an independent predictive factor. The presence of infection was set as the dependent variable, and the significantly regulated parameters according to the patients’ demographic characteristics (NIHSS and SAA) were set as confounders. WBC and CRP were also included in the confounder group because they are widely used in clinical practice. As Table 3 shows, SAA was the only marker that displayed a relationship with the development of post-stroke infections, thus confirming and validating the possibility of measuring SAA concentrations as a biomarker of infection in stroke patients.

**Table 3:** Multivariate analyses of different factors predicting the presence of infection

Infection			
Predictors	OR	95% CI	*p*
SAA	3.68	(1.27–10.6)	0.047
CRP	0.37	(0.12–1.17)	0.09
WBC	1.24	(0.86–1.79)	0.24
NIHSS	1.18	(1.01–1.38)	0.031

## Discussion

The present study highlighted the capacity of proteomics to identify protein biomarkers that could assist in the detection of stroke patients at a high risk of developing post-stroke infection [34]. Using isobaric labeling methods, we first compared the plasma samples of five infected stroke patients and five non-infected stroke patients. We found that concentrations of serum amyloid A1 were overexpressed in patients who went on to develop an infection. This first approach was then verified using parallel reaction monitoring in 20 stroke patients (10 infected and 10 non-infected). Finally, the SAA1/2 concentrations of 40 ischemic stroke patients were confirmed using ELISA kits. The results demonstrated that SAA1/2 is an efficient infection-risk prediction marker in stroke patients.

SAA1/2 has already been described as a potential marker of inflammation and infection in several pathological conditions, including stroke and subarachnoid hemorrhage, [35]. In a case-controlled study involving 54 patients, levels of acute-phase proteins were significantly higher in stroke patients who developed an infection during the month preceding a cerebrovascular event than in those who did not develop one. During the month following the stroke, concentrations of SAA started being significantly higher in patients who went on to develop an infection than in those who had only had an infection preceding the cerebrovascular event at 3 days after the onset of symptoms [36]. This highlighted that the acute-phase response was clearly related to the development of infections. In another study, of 60 patients, levels of SAA were significantly higher in 45 patients with stroke than in the 15 control patients without stroke. SAA concentrations increased between days one and three in patients with a cerebral infarction complicated by an infectious inflammatory process [37]. These results suggested a correlation between the acute-phase response and the development of an infection. Nevertheless, to the best of our knowledge, until now no one had evaluated the ability of these acute-phase molecules to act as predictors of infection.

In a population of 81 subarachnoid hemorrhage patients, SAA concentrations measured at hospital admission predicted which patients would develop an infection during their hospital stay with an accuracy of 76% [38]. We therefore decided to perform the same analysis using ischemic stroke patients in the present study. As already shown, very similar results were obtained.

To the best of our knowledge, our study is the first to assess the predictive value of SAA concentrations while taking into account the time points of measurements as well as the diagnosis. We found that the SAA concentration was able to detect 42.9% of the stroke patients who had a very high certainty of going on to develop an infectious complication.

Human SAA is an acute-phase protein primarily expressed by the liver [39]. There are four different but closely related genes responsible of the protein’s different isoforms. In humans, the production of SAA1 and SAA2 takes place under inflammatory conditions. SAA3 is a pseudo-gene, and SAA4 encodes a protein that is produced constitutively [40]. Inflammatory SAA1 and SAA2 share around 90% of their gene sequence. Due to the similarities between both, immunoassays have been unable to differentiate between them [39], and studies to date have been unable to determine which of the isoforms is most associated with infectious and inflammatory processes. In the present study, we used the PRM method to track each isoform and evaluate its contribution. As shown in Additional file 2: Fig. 1, both SAA1 and SAA2 are related to infection development. The FFGHGAEDSLADQAANEWGR peptide, unique to SAA1, and the tryptic SAA2 GPGGAWAAEVISNAR peptide appeared to be significantly more abundant in patients suffering from an infection than in patients without one.

SAA concentrations are most likely higher in stroke patients suffering from infections due to its role in attracting leukocytes and immune cells to the sites of tissue damage, infection, or inflammation [41, 42]. As previously described, inflammation is an important part of the reactions taking place after an ischemic event. Indeed, blood derived leukocytes and microglia will be activated from minutes to hours after a cerebrovascular event [43]. Recruitment, activation, and adhesion of leukocytes to the endothelium will happen at the same time as neutrophils and monocytes/macrophages transmigrate into the location of the cerebral infarction [44]. During this process of brain damage, the acute-phase response will also activate acute-phase proteins as SAA, CRP, haptoglobin, α1-acid glycoprotein, α1-antichymiotripsin appear increasingly in the blood [44].

The present study has certain limitations. (1) The cohort was small and its results should be validated in a larger cohort of patients in order to have sufficient samples for the subgroup analyses (infection, no-infection). (2) The study proposed SAA as a promising prognostic infection marker in stroke patients. Nevertheless, combining SAA concentrations with other clinical scales (NIHSS) or scores could improve the accuracy of the association. Different combinations should be tested to evaluate the potential added value of a panel of markers. (3) Another point which remains to be investigated is why SAA concentrations become elevated in patients developing an infection much earlier than CRP does, for example. The present study postulated that this was due to its role in inflammation, but are we thus measuring inflammation or are we facing a post-infection inflammation phenomenon? As previously reported, the acute-phase response is more prominent in patients who develop an infection during hospitalization, but SAA concentrations were already higher when a previous bacterial infection was present. A detailed study should be performed to compare all these factors. (4) Different isoforms of SAA did not seem to act differently in conditions of inflammation. Nevertheless, the present study was only able to measure one tryptic peptide from each isoform. Further studies should target different peptides corresponding to the different isotypes of each isoform in order to perform a give a more detailed analysis of the role of SAA in infected stroke patients. Finally, to translate this study’s results into clinical practice, a point of care test should be developed in order to provide results in minutes and ensure better, faster patient management.

## Conclusions

In a small cohort of stroke patients, we were able to demonstrate that the concentrations of SAA1/2 measured at hospital admission could be used to predict post-stroke infection. Applying SAA measurement in clinical settings could drastically improve patient management and, consequently, their associated outcomes. Further large, multicenter validation studies are needed to confirm these results.

## Additional files

**Additional file 1.** List of proteins identified when comparing the proteomes of five infected and five non-infected patients.

**Additional file 2.** Ratio of FFGHGAEDSLADQAANEWGR and GPGGAWAAEVISNAR peptide abundance between patients who went on to develop an infection and those who did not.

**Additional file 3.** Kinetics of SAA concentrations at hospital admission, 1 day, 3 days and 5 days after stroke onset, including discovery step patients only (a) and verification step patients only (b).

## Abbreviations

AUC: area under the ROC curve
A2DS2: age, atrial fibrillation, dysphagia, sex, stroke severity
CDC: Centers for Disease Control and Prevention
CI: confidence interval
CID: collision-induced dissociation
CRP: C-reactive protein
CSF: cerebrospinal fluid
CT: computed tomography
ELISA: enzyme-linked immunosorbent assay
ECG: electrocardiogram
FDR: false discovery rate
HCD: higher-energy collisional dissociation
HPLC: high-performance liquid chromatography
HRP: horseradish peroxidase
IQR: interquartile range
IPG: immobilized pH gradient
MSD: mesoscale discovery
NIHSS: National Institutes of Health Stroke Scale
OGE: off-gel electrophoresis
OT: OrbiTrap
PRM: parallel reaction monitoring
ROC: receiver operating characteristic
PCT: procalcitonin
SAA1/2: serum amyloid 1/2
SE: sensitivity
SP: specificity
TEAB: tetraethylammonium bromide
TCEP: tris-(2-carboxyethil) phosphine hydrochloride
TMT: tandem mass tag
TOAST: trial of org 1072 in acute stroke treatment
WBC: white blood cells
