# Supplementary material for: Proteomic discovery and verification of serum amyloid A as a predictor marker of patients at risk of post-stroke infection: a pilot study
Source: Clin Proteomics. 2017 Jul 12;14:27. doi: 10.1186/s12014-017-9162-0 (PMC5506582; doi:10.1186/s12014-017-9162-0)
Supplement: Supplementary file 2 — Additional file 2. Ratio of FFGHGAEDSLADQAANEWGR and GPGGAWAAEVISNAR peptide abundance between patients who went on to develop an infection and those who did not. [file 12014_2017_9162_MOESM2_ESM.pdf]

Supplementary figure 1: FFGHGAEDSLADQAANEWGR and GPGGAWAAEVISNAR peptide abundance ratio in patients who will or will not develop infections.

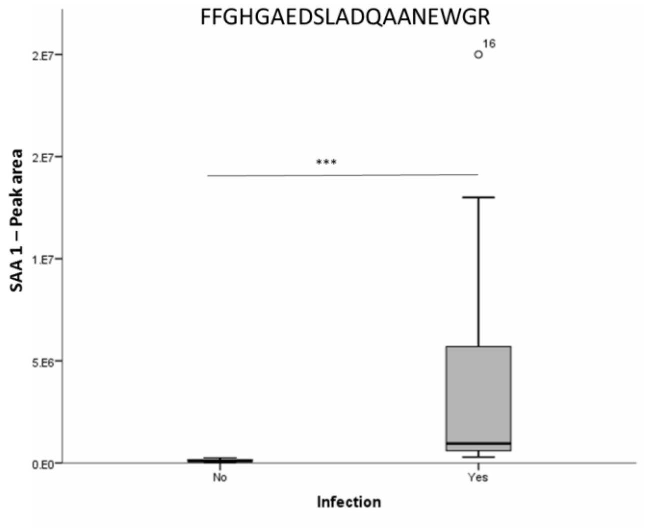

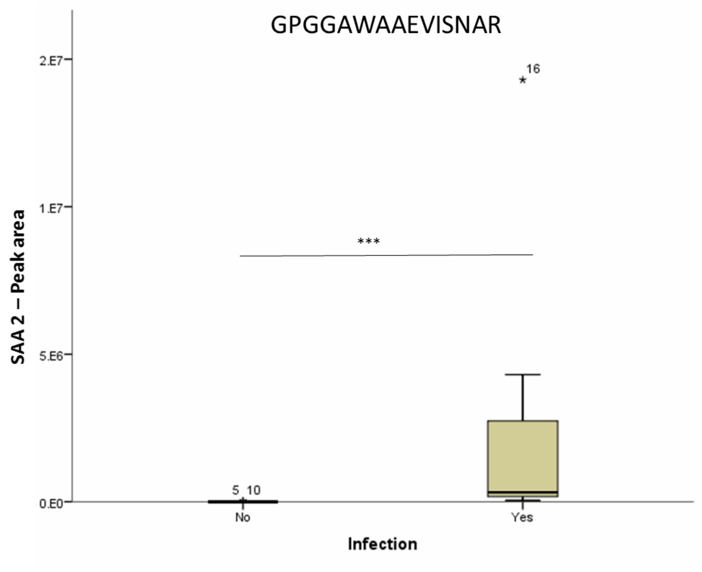

The peptide FFGHGAEDSLADQAANEWGR is proteotypic of SAA1 and GPGGAWAAEVISNAR is proteotypic of SAA2.
