# Supplementary material for: Proteomic discovery and verification of serum amyloid A as a predictor marker of patients at risk of post-stroke infection: a pilot study
Source: Clin Proteomics. 2017 Jul 12;14:27. doi: 10.1186/s12014-017-9162-0 (PMC5506582; doi:10.1186/s12014-017-9162-0)
Supplement: Supplementary file 3 — Additional file 3. Kinetics of SAA concentrations at hospital admission, 1 day, 3 days and 5 days after stroke onset, including discovery step patients only (a) and verification step patients only (b). [file 12014_2017_9162_MOESM3_ESM.pdf]

Supplementary figure 2: Kinetics of SAA concentrations at hospital admission, 1 day, 3days and 5 days after stroke onset including only discovery step patients (a) and only verification step patients (b).

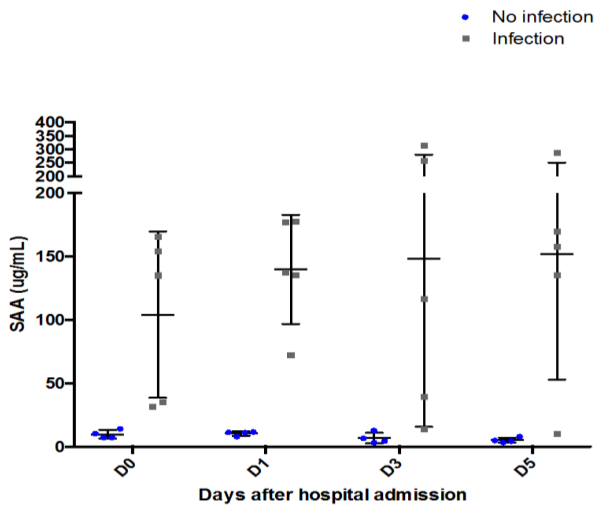

Discovery patients

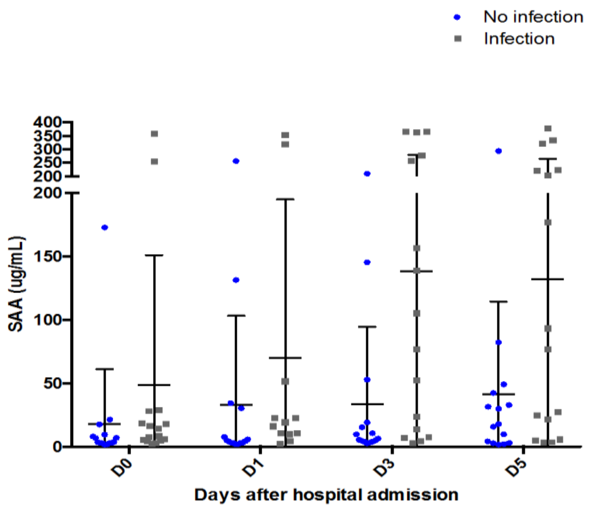

Verification patients
